# The Prevalence of Physical Intimate Partner Violence During Pregnancy and the Postpartum Period: A Systematic Review With Implications for Probable Violence-Caused Brain Injury Among Child Bearers

**DOI:** 10.1177/15248380241309292

**Published:** 2025-01-02

**Authors:** Shambhu P. Adhikari, Tori N. Stranges, Bradi R. Lorenz, Rory A. Marshall, Nelson Jiang, Paul van Donkelaar

**Affiliations:** 1 University of British Columbia, Kelowna, Canada; 2 University of Western Ontario, Canada

**Keywords:** brain injury, domestic violence, intimate partner violence, postpartum, pregnancy, prevalence

## Abstract

Intimate partner violence (IPV) persists as a cause of short-term, long-term, and chronic health consequences. The elevated risk of IPV during pregnancy and the postpartum period (P-IPV) is commonly attributed to increased demands for child bearers and intimate partners. P-IPV may impact the health of the child bearer, developing fetus, and post-birth child. The prevalence of physical P-IPV remains under-explored. The primary objective of the study is to describe the prevalence of physical P-IPV during the period from pregnancy through 24 months postpartum. *Medline* (*PubMed*), *Embase*, *CINAHL*, and *PsycINFO* were searched (2000–2023) using the PICO model, MeSH terms, and Boolean operators. Studies with intimate partners exposed to physical IPV during pregnancy and the postpartum period that described the prevalence of IPV were included. Fifty-five studies were included. The sample-weighted average prevalence of physical P-IPV was calculated as 14.7% (range 0.6%–52.4%, *n* = 55). The sample-weighted average prevalence of physical IPV during pregnancy was calculated as 4.4% (0.6%–42.5%, *n* = 48). The sample-weighted average prevalence of physical IPV during the postpartum period was calculated as 10.3% (2.2%–52.4%, *n* = 16). The prevalence of physical P-IPV remains a looming threat to child bearer, fetal, and early childhood health. Given the >80% prevalence of IPV-caused brain injury (IPV-BI) from physical IPV, brain injury is likely occurring during pregnancy and the postpartum period and must be considered. Further investigations should be undertaken to uncover the true prevalence and impact of BI during this timeframe and mitigate the risk of P-IPV.

## Background

Intimate partner violence (IPV; i.e., dating violence, partner abuse, and spousal abuse) is defined as physical, sexual, financial, and/or psychosocial abuse perpetrated by a current or former intimate partner (World Health Organization [WHO], 2021). IPV affects individuals regardless of socioeconomic status, religious affiliation, geographic location, race, ethnicity, sexual orientation, and/or gender identity (Scheer et al., 2022). Research estimates that the lifetime prevalence of IPV among women is 1 in 3, and the 12-month prevalence is 1 in 10 (Black et al., 2011; WHO, 2018). Physical IPV is defined as any intentional use of physical force with the potential for causing death, injury, or harm (WHO, 2021). This form of violence encompasses a range of behaviors, including hitting, punching, kicking, slapping, and other forms of physical aggression. Physical IPV leads to a vast range of psychological and physical health consequences. Acute physical injury manifestations commonly evolve into or are accompanied by IPV-caused conditions, including headaches, chronic pain, pelvic pain, heart palpitations, anxiety, irritable bowel syndrome, vaginal infections, and cardiovascular, urological, gynecological, and pregnancy-related conditions (Stubbs & Szoeke, 2022). Given the spectrum of IPV assaults, corresponding injuries vary from minor bruises and abrasions, multisystem trauma, or death (Sun et al., 2022).

The pregnancy (conception through childbirth) and postpartum (childbirth through 24 months post-childbirth) period (Mojahed et al., 2021) has been shown to increase the risk for child bearing people to experience IPV due to increased physical and mental vulnerability during this time resulting from higher demands on individual capacities, intimate relationships, and household economic resources (Burke et al., 2008; Fisher et al., 2012; O’Shea et al., 2016). As such, IPV during pregnancy and the postpartum period (P-IPV) is a leading cause of death and injury for women of reproductive age (Kapaya et al., 2019). Exposure to P-IPV involves serious risks of adverse maternal and natal outcomes. For child bearers who are exposed to IPV, there is an increased likelihood their child will develop behavioral problems including anxiety, depression, aggressive behavior, attention problems, and overall scores for internalizing and externalizing behavioral problems at 12, 24, and 36 months of age (Fredland et al., 2018; Letourneau et al., 2019). Further, children born to those exposed to P-IPV are at risk for adverse developmental disabilities and congenital defects such as language and neurological delay, decreased motor development, and reduced stress regulation (Davis et al., 2011; Grace et al., 2016; Udo et al., 2016). Physical IPV targeting a child bearer’s abdomen during pregnancy poses significant risks to the fetus, including miscarriage, preterm birth, low birthweight, and stillbirth (Alhusen et al., 2015; Hill et al., 2016; Silverman et al., 2006; Tiwari et al., 2008). Further, IPV often persists during pregnancy and the postpartum period, increasing the child bearer’s risk for postpartum depression, anxiety, and PTSD, which may impair parenting (Chiesa et al., 2018; Pastor-Moreno et al., 2020). The vulnerability of the pregnancy and postpartum period underscores the need for targeted interventions to mitigate the extensive health impacts of IPV during this time.

Research and public health efforts have highlighted that those who experience physical IPV may also experience an IPV-caused brain injury (IPV-BI) (Campbell et al., 2022; Esopenko et al., 2021; Haag et al., 2022; Valera & Berenbaum, 2003). Amid physical IPV, IPV-BI occurs in over 80% of cases (Esopenko et al., 2021). IPV-BI commonly occurs from direct blows (e.g., punches, kicks, slaps), indirect force (e.g., being thrown into a wall, being pushed down the stairs), and/or non-fatal strangulation (NFS; e.g., being “choked out”) (Esopenko et al., 2021; Valera & Berenbaum, 2003). BIs are well documented as causing short- and long-term health consequences independent of the detriment of experiencing IPV. At this time, minimal research currently exists at the intersection of P-IPV and IPV-BI.

P-IPV is an ongoing and growing concern worldwide. Furthermore, physical P-IPV poses increased health risks above other forms of P-IPV alone. Establishing a current estimation of physical P-IPV prevalence in the current global context is necessary as research indicates that IPV has increased substantially in the wake of coronavirus disease of 2019 (COVID-19) (Gilchrist et al., 2023). Given this global change affecting different regions in varying manners, this systematic review aims to evaluate the literature to describe a prevalence estimate of physical P-IPV.

## Objective

The objective of this systematic review was to investigate the current prevalence of physical P-IPV, across the pregnancy (conception through childbirth) and postpartum (childbirth through 24 months post-childbirth) period. Following the initial review, post hoc secondary objectives were identified to examine (a) prevalence during pregnancy and the postpartum period, (b) regional prevalence, (c) prevalence across socioeconomic status, and (d) prevalence in relation to COVID-19.

## Methods

### Search Strategy

A systematic search was performed in August 2023 (to search articles published between January 2000 and July 2023) through four different databases: *Medline* (*PubMed*) via Ovid, *Embase* via Ovid, *CINAHL* via EBSCO, and *PsycINFO* via EBSCO. The search strategy was developed according to the PIO (Population, Issue, Outcome) framework (Akobeng, 2005; Calderon Martinez et al., 2023) to determine search concepts and types of studies to include (Supplemental materials). Separate searches were conducted for each primary database. The Medical Subject subHeadings (MeSH) terms and keywords were combined with the Boolean operators (AND) and (OR). Various commands were employed to enable multiple spellings and positionings of key terms, which were then mapped to relevant subject headings and search fields. The keywords (and their combinations) adopted for the research are the following: Perinatal OR Perinatal period OR puerperium OR postpartum OR postpartum period OR Prenatal OR prenatal period OR postnatal OR postnatal period OR peripartum OR peripartum period OR pregnancy OR pregnant OR pregnant women AND Intimate partner violence OR IPV OR intimate partner abuse OR partner violence OR Partner abuse OR domestic violence OR spouse abuse OR marital rape OR domestic abuse OR battered women OR battered wife OR battered females OR gender-based violence OR gender violence OR physical abuse OR dating violence OR dating abuse OR elder abuse AND Prevalence. The full list of search terms and search strategy are presented in Supplemental materials.

To capture relevant literature that met the inclusion criteria but was not accessible through the listed databases, gray literature (defined as “the documents produced on all levels of governments, academics, business, and organizations in electronic or print format not controlled by commercial publishers”) (Mahood et al., 2014; Paez, 2017), sources were sought using internet search engines (e.g., *Google*, *Google Scholar*), forward citation searching (examining articles that are citing an article being observed), reference checking (also backward citation searching), and expert consultation with a librarian (Korab-Chandler et al., 2022; Paez, 2017). Using these search strategies, pre-print articles, theses, dissertations, conference proceedings, evidence-based organizational reports, government reports, etc., were sorted and evaluated for eligibility in the inclusion process (Paez, 2017).

### Inclusion Process

The inclusion criteria included original sources that (a) reported prevalence of physical IPV that occurred during the pregnancy and postpartum period, (b) were published between January 2000 and July 2023, and (c) had full texts available in English. The exclusion criteria were (a) review articles, meta-analysis, case study, or case reports, and (b) qualitative studies.

**Figure 1.**
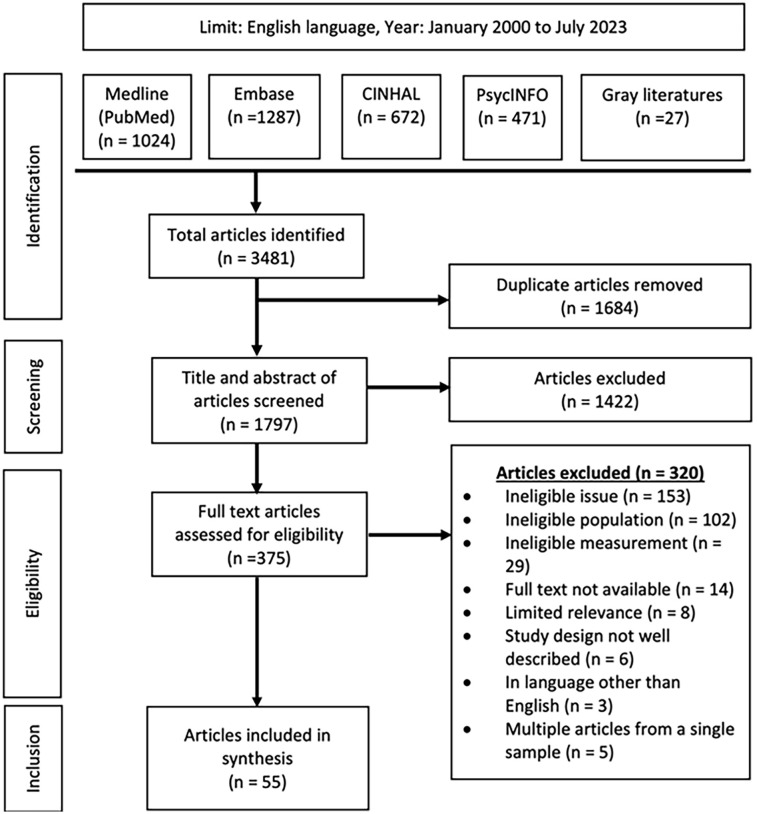
PRISMA.

A PRISMA flowchart of the search strategy and inclusion process can be seen in Figure 1. Covidence (Veritas Health Innovation, Melbourne, Australia; University of British Columbia licensed version) was used for the search strategy compilation, duplicate removal, title and abstract screening, and full-text review. The search strategies yielded 3,454 results, and the gray literature search yielded 27 results. Once duplicates were removed from the total of 3,481 articles, 1,797 articles were advanced to the title and abstract screening phase. Two independent reviewers (SPA and NJ) screened the titles and abstracts against the inclusion and exclusion criteria. The two reviewers had an alignment rate of over 95% (1,719/1,797). For title and abstract screening conflicts, a consensus was reached following discussion among SPA, NJ, and where appropriate a third reviewer (TNS).

In total, 375 articles were advanced to the full-text review phase. Full texts were reviewed independently (SPA and TNS) against the inclusion and exclusion criteria. The two reviewers had an alignment rate of over 98% (370/375). For full-text review conflicts, a consensus was reached following discussion among SPA, TNS, NJ, and where appropriate a fourth reviewer (PvD). Ultimately, 55 articles examining the prevalence of physical P-IPV were included in this review.

### Data Extraction and Synthesis

Three independent team members (SPA, TNS, and BRL) critically appraised, extracted, categorized, and synthesized data into separate Excel spreadsheets. Data extraction sheets were uniformly formatted for easy reference and relevant comparison. This data was then audited for accuracy and consistency by the research team. A majority consensus was used to confirm accuracy. This verified outcome data was advanced to the data analysis stage.

### Quality Assessment of the Studies Included in the Review

The Joanna Briggs Institute (JBI) critical appraisal checklist for studies reporting prevalence data (Joanna Briggs, 2017) was chosen for the quality assessment of the studies included in this review. The JBI appraisal checklist was selected based on specialist librarian guidance and evidence of its use in reviews estimating the prevalence of IPV (Nepal et al., 2023; Schrubbe et al., 2023). A higher score on the JBI checklist indicates the authors more closely adhered to methodological best practices outlined in the JBI critical appraisal checklist (Joanna Briggs, 2017). The article's JBI score included in the review is presented in Table 1. No studies were excluded from this review based on the JBI score.

**Table 1.** Characteristics of the Studies Included in the Review.

Articles	Quality (JBI) [9]	Country	Data Collection Period Related to COVID-19	Study Design and Setting	Sample Size	Measurement Used for Documenting Violence	Prevalence of IPV	Prevalence of Physical IPV
Abota et al. (2022)	9	Ethiopia	Pre-COVID-19	Cross-sectional study, Community-based data	1,292	WHO	P: 28.3% PP: 22.4%	P: 17.0% PP: 13.0%
Adhena et al. (2020)	9	Ethiopia	Pre-COVID-19	Cross-sectional study, Public Health Facility-based data	538	WHO	P: 37.5%	P: 13.4%
Ahinkorah et al. (2023)	8	Africa (Sub-Saharan countries)	Pre- and during COVID-19 (2010–2020)	Cross-sectional database study, Demographic and Health Surveys, 2010–2020	108,971	WHO as part of DHS Global Survey	NA	P: 6.0%
Amir et al. (2018)	9	Iran	Pre-COVID-19	Cross-sectional study, Public Health Centre-based data	398	Revised CTS-2	P: 58.0%	PP: 33.0%
Atilla et al. (2023)	8	Turkey	During COVID-19	Cross-sectional study, Hospital-based data	456	Intimate Partner Violence During Pregnancy Instrument	N/A	P: 6.6%,
Ayodapo et al. (2017)	9	Nigeria	Pre-COVID-19	Cross-sectional study, Health Care Facility-based data	350	WHO	P: 17.4%	P: 21.4%
Azene et al. (2019)	9	Ethiopia	Pre-COVID-19	Cross-sectional study, Public Health-based data	418	Adapted WHO	P: 41.4%	P: 21.0%
Belay et al. (2019)	8	Ethiopia	Pre-COVID-19	Cross-sectional study, Community-based data	589	Adapted WHO	P: 21.0%	P: 9.2%
Belay et al. (2022)	8	Ethiopia	During COVID-19	Cross-sectional study, Facility-Based data	657	Adapted WHO	P: 62.4%	P: 21.3% PP: 9.7% P&PP: 3.8%
Bikinesi et al. (2017)	8	Namibia	Pre-COVID-19	Descriptive survey, Primary Care Clinic-based data	386	Adapted WHO	P: 8.0%	P: 3.4%
Boah et al. (2023)	8	Ghana	During COVID-19	Cross-sectional study, Public Health Facility-based data	402	WHO as part of DHS Global Survey	P: 35.1%	P: 6.7%
Castro et al. (2003)	7	Mexico	Pre-COVID-19	Comparative study, Prenatal Clinic-based data	914	CTS-2	P: 24.5%	P: 10.0%
Charles et al. (2007)	8	United States	Pre-COVID-19	Stratified database study, Hospital-based data	2310	The Future of Families and Child Wellbeing Study *Previously Fragile Families Study	P: 19.8% PP: 51.7%	P: 9.6% PP: 3.1%
D’Angelo et al. (2023)	9	United States	Pre-COVID-19	Cross-sectional database survey, 2016–2018	15,592	PRAMS	P: 5.7%	P: 1.5%
Edhborg et al. (2020)	8	Bangladesh	Pre-COVID-19	Longitudinal study, Organization-based data	656	Adapted WHO	PP: 89.0%	PP: 52.0%
Eikemo et al. (2023)	9	Sweden	During COVID-19	Cross-sectional Survey, Clinic-based data	3,371	AAS	P: 2.1%	P: 0.6%
Ezeudu et al. (2019)	7	Nigeria	Pre-COVID-19	Cross-sectional study, Population-based data	702	86-item interviewer administered semi-structured questionnaire	P: 37.2%	P: 42.5%
Farrokh-Eslamlou et al. (2014)	9	Iran	Pre-COVID-19	Cross-sectional study, Hospital-based data	313	Adapted AAS	P: 55.9%	P: 10.2%
Fekadu et al. (2018)	9	Ethiopia	Pre-COVID-19	Cross-sectional study, Clinic-based data	450	WHO Multi-Country Study (WHO) questionnaire	P: 58.7%	P: 32.2%
Fetene et al. (2022)	9	Ethiopia	During COVID-19	Cross-sectional study, Community-based data	590	WHO with some contextual modifications	P: 39.3%	P: 29.8%
FitzPatrick et al. (2022)	9	Australia	Pre-COVID-19	Prospective cohort study, Hospital-based data	1,346	Composite Abuse Scale (18-item version)	PP: 17.4%	PP: 2.2%
Fonseca-Machado et al. (2015)	8	Brazil	Pre-COVID-19	Cross-sectional study, Hospital-based data	358	Adapted WHO	P: 17.6%	P: 6.4%*
Gebrekristos et al. (2023)	8	South Africa	Pre-COVID-19	Cross-sectional pilot study, Hospital-based data	90	10 items (on physical & psychological violence) from the WHO and CTS-2	P: 40.0%	P: 16.7%
Gebrezgi et al. (2017)	9	Ethiopia	Pre-COVID-19	Cross-sectional study, Public Health Facility-based data	422	WHO	NA	P: 20.6%
Guo et al. (2004)	7	China	Pre-COVID-19	Survey, Community-based data	12,044	The survey assessed various forms of violence using different questions.	P: 3.6% PP: 7.4%	P: 1.3% PP: 3.8%
Haight et al. (2022)	9	Pakistan	Pre-COVID-19	Longitudinal study, Organization-based data	815	Adapted WHO	PP: 50.6%	PP: 9.3% **
Haron et al. (2021)	9	Malaysia	Pre-COVID-19	Cross-sectional study, Hospital-based data	1,200	WHLE—10th version	P: 35.9%	P: 12.9%
Hedin et al. (2000)	7	Sweden	Pre-COVID-19	Cross-sectional study, Community-based data	132	SVAW	P: 24.2%	P: 9.9%
Hossieni et al. (2017)	9	Iran	Pre-COVID-19	Cross-sectional study, Health Centre-based data	174	CTS-2	P: 73.0%	P: 23.0%
Ibrahim et al. (2015)	8	Egypt	Pre-COVID-19	Prospective cohort study, Hospital-based data	1,857	The NorVold Domestic Abuse Questionnaire	P: 44.1%	P: 15.9%
Islam et al. (2021)	9	Bangladesh	Pre-COVID-19	Retrospective cross-sectional survey, Clinic-based data	426	Adapted WHO	P: 66.4% PP: 63.6%	P: 35.2% PP: 32.2%
Jackson et al. (2015)	8	United States	Pre-COVID-19	Cross-sectional study, Clinic-based data	320	PRAMS	P: 11.3%	P: 3.4%
Jungari et al. (2022)	9	India	Pre-COVID-19	Cross-sectional study, Community-based data	500	Adapted WHO	P: 15.6%	P: 9.2%
Kabir et al. (2014)	7	Bangladesh	Pre-COVID-19	Part of a longitudinal study, Community-based data	660	Adapted WHO	NA	P: 18.0% PP: 52.0%
Kapaya et al. (2019)	8	United States	Pre-COVID-19	Mixed-mode population-based surveillance system, Population-based data	254,462	PRAMS	NA	P: 2.6%
Kataoka et al. (2016)	8	Japan	Pre-COVID-19	Brief report of a cross-sectional survey, Hospital-based data	82	SVAW	P: 20.7%	P: 3.7%
Lencha et al. (2019)	8	Ethiopia	Pre-COVID-19	Cross-sectional study, Hospital-based data	612	Adapted WHO	P: 59.0%	P: 20.3%
Makayoto et al. (2013)	8	Kenya	Pre-COVID-19	Cross-sectional study, Hospital-based data, 2010	300	The questionnaire included variables related to social and demographic characteristics of the pregnant women	P: 37.0%	P: 26.0%
Moraes et al. (2017)	8	Brazil	Pre-COVID-19	Cross-sectional study, Primary care clinic-based data	1,082	CTS-2	N/A	PP: 18.3%
Munro-Kramer et al. (2018)	8	Zambia	Pre-COVID-19	Quantitative household survey, Rural health facility-based data	2,381	2-item physical IPV Likert scale	N/A	PP: 8.1%
Musa et al. (2020)	9	Ethiopia	Pre-COVID-19	Cross-sectional survey, Hospital-based data	648	Adapted WHO	PP: 39.8%	PP: 25.9%
Okunola et al. (2021)	7	Nigeria	Pre-COVID-19	Prospective Cohort Study, Hospital-based data	363	Ongoing abuse screen	PP: 15.4%	P: 3.6%
Onoh et al. (2013)^†^	8	Nigeria	Pre-COVID-19	Cross-sectional survey, Antenatal Clinic-based data	321	Pre-tested semi-structured questionnaire	P: 44.6%	P: 21.0%
Onuonga et al. (2023)	9	Kenya	During COVID-19	Cross-sectional survey, Prenatal care clinic-based data	360	WHO	P: 35.3%	P: 15.6%
Rurangirwa et al. (2017)	9	Rawanda	Pre-COVID-19	Cross-sectional study, Population-based data	922	WHLE	P: 19.2%	P: 10.2% PP: 12.1%
Scribano et al. (2013)	8	United States	Pre-COVID-19	Database study, NFP database	10,855	AAS	N/A	P: 8.1% PP: 12.4%
Shamu et al. (2013)	9	Zimbabwe	Pre-COVID-19	Cross-sectional survey, Primary health care-based data	2,024	Adapted WHO	P: 63.1%	P: 15.9%
Silva et al.(2011)	9	Brazil	Pre-COVID-19	Prospective cohort interview study, Family Health Program-based data	960	WHO	P: 31.0% PP: 22.6% EPP: 47.4%	P: 11.6% PP: 12.1%
Silva et al. (2020)	8	Brazil	Pre-COVID-19	Cross-sectional interview, Maternity Hospital-based data	330	WHO	N/A	P: 7.6%
Thomas et al. (2019)	9	United States	Pre-COVID-19	Structure Interviews, Community Health Centre & Obstetric Outpatient Clinic-based data	930	CTS-2	P: 38.8%	P: 37.5%
Umoh et al. (2012)	6	Nigeria	Pre-COVID-19	Cross-sectional study, Antenatal Clinic-based data	442	Questions about physical assault by their spouse during pregnancy	N/A	P: 10.3%
Utaile et al. (2023)	9	Ethiopia	During COVID-19	Cross-sectional Survey, Household community-based data	1,435	WHO	P: 48.0%	P: 24.6%
Valladares et al. (2005)	9	Nicaragua	Pre-COVID-19	Cross-sectional survey, Community-based data	478	WHO	P: 32.0%	P: 13.4%
Verma et al. (2007)	9	India	Pre-COVID-19	Cross-sectional interviews, Antenatal Clinic-based data	203	Index of Spouse Abuse	N/A	P: 1.0%
Wood et al. (2022)	8	Ethiopia	Pre- and during COVID-19	Cohort study (multimethod), Data from Performance Monitoring for Action Ethiopia	2,868	CTS-2	P (pre-COVID-19): 10.5% P (during COVID-19): 15.1%	P (pre-COVID-19): 5.0% P (during COVID-19): 8.0%

*Note*. P = pregnancy; PP = postpartum, P&PP = pregnancy and postpartum; WHO = World Health Organization Multicounty Study; PRAMS = pregnancy risk assessment monitoring system; DHS = demographic and health surveys; CTS-2 = conflict tactic scale-2; WHLE = women’s health and life experiences questionnaire; SVAW = severity of violence against women scale; NFP = nurse family partnership; IPV = intimate partner violence; AAS = abuse assessment screen. *The prevalence of physical IPV is 6.4% (23 out of 358); **the prevalence of physical IPV at 12 months postpartum (8.5%) is mentioned only in the abstract whereas the prevalence at 24 months postpartum (9.3%) is presented in the results section, so we considered 9.3% as the prevalence of postpartum physical IPV for this review; ^†^The prevalence of physical IPV is differently presented in this article (physical and/or emotional is documented in the results section whereas physical and emotional is documented in the abstract and discussion sections). The authors included this article in this review considering the data documented is the prevalence of physical and emotional IPV.

### Reporting Prevalence and Data Visualization

Owing to the small number of studies spanning the pregnancy and postpartum period and in an attempt to generate the most accurate estimate based on the most data, physical P-IPV prevalence rates weighted to sample size were calculated to derive sample-weighted averages (Maidment et al., 2014; Sheeran et al., 2020) separately during the pregnancy period and the postpartum period. The sample-weighted averages for the pregnancy period and the postpartum period were summed to estimate the prevalence of physical P-IPV.

For all other analyses, studies were grouped according to their reported outcome measures and the context of the post hoc sub-analysis. Forest plots were generated to summarize and visualize the prevalence of physical P-IPV findings (Stata 14, College Station, TX, USA). The effect size-weighted averages for prevalence by group were generated during each forest plot analysis using a random effects model (Marlborough et al., 2021; Watters et al., 2019). The articles included in the review were organized into different sub-groups as follows:

1. Socioeconomic classification: The prevalence by socioeconomic classification was categorized in alignment with the World Bank (low-income countries, lower-middle-income countries, upper-middle-income countries, and high-income countries) (World-Bank, 2022).
2. Regions: The prevalence by region was categorized in alignment with the WHO (Asia, Africa, Europe, Americas, and Oceania) (Devries et al., 2010). In reference to the Pan-American Health Organization and WHO, all the American countries were grouped under “Americas.”
3. COVID-19: The prevalence relative to the COVID-19 pandemic was classified in alignment with the WHO declarations of the beginning (March 11, 2020) and end of the public health emergency (May 5, 2023), as pre-pandemic (prior to March 2020), during pandemic (March 11, 2020-May 2023), and post-pandemic (May 2023 onward) (WHO, 2023). Thus, the articles included in this review were grouped into pre-COVID-19 (data collected before March 2020) or during COVID-19 (data collected between March 2020 and May 2023). None of the articles included in this review recruited participants post-COVID-19 (after May 2023).

## Results

### Extraction Information and Analysis of the Article

The results of the search strategies (Supplemental Tables 1 and 2) yielded a total of 3,489 articles. After the eligibility screening process, 55 articles were included in this systematic review (Figure 1). Of note, none of the articles that were found through gray literature search strategies met the inclusion criteria. Included studies that defined physical P-IPV in a variety of ways using several different assessment tools (Supplemental Table 3). In addition to physical P-IPV prevalence, P-IPV prevalence (in general, not just physical) was reported in 44 (80.0%) studies (Table 1).

### General Characteristics of Articles Included in the Review

Out of 55 articles included in the review, 37 (67.3%) studies were either hospital-based, clinic-based, or health facility-based (Table 1), 10 (18.2%) studies were conducted in the community, and eight (14.5%) studies analyzed either population-based or organization-based data.

Out of 55 articles included in the review, 27 (49.1%) studies used the WHO multi-country study questionnaire for the assessment of physical violence (Supplemental materials**)**. Furthermore, only two (3.6%) studies were longitudinal. The remaining 53 (96.4%) studies were either cross-sectional or prospective cohort studies. Participants recruited in all the studies were heterogeneous in terms of demographic characteristics such as age, education, or marital status.

Out of 55 articles included in the review, 39 (70.9%) reported the prevalence of physical IPV during pregnancy, seven (12.7%) articles reported the prevalence of physical IPV during postpartum period, eight (14.6%) reported the prevalence of physical P-IPV, and one article (1.8%) reported the prevalence of physical IPV separately for the pregnancy period and the postpartum period, in addition to the prevalence of physical P-IPV, which was 3.8% (Belay et al., 2022).

Out of the 55 studies, 46 (83.7%) recruited participants before COVID-19, seven (12.7%) recruited participants during COVID-19, and one study (1.8%) recruited participants before and during COVID-19 pandemic. The one study (Ahinkorah et al., 2023) that recruited participants before and during the COVID-19 pandemic (survey years ranging from 2010 to 2020) did not report separately for the two time periods, and thus, this study was excluded in calculating the prevalence of IPV based on time period related to COVID-19 pandemic.

### Overall Physical P-IPV Prevalence

The sample-weighted average prevalence of physical P-IPV was 14.7% (overall range 0.6%–52.4%, *n* = 55, summed pregnancy and postpartum period average; pregnancy, sample-weighted average 4.4% [0.6%–42.5%, *n* = 48]; postpartum period sample-weighted average 10.3% [2.2%–52.4%, *n* = 16]).

### Prevalence of Physical IPV During Pregnancy and the Postpartum Period

**Figure 2.**
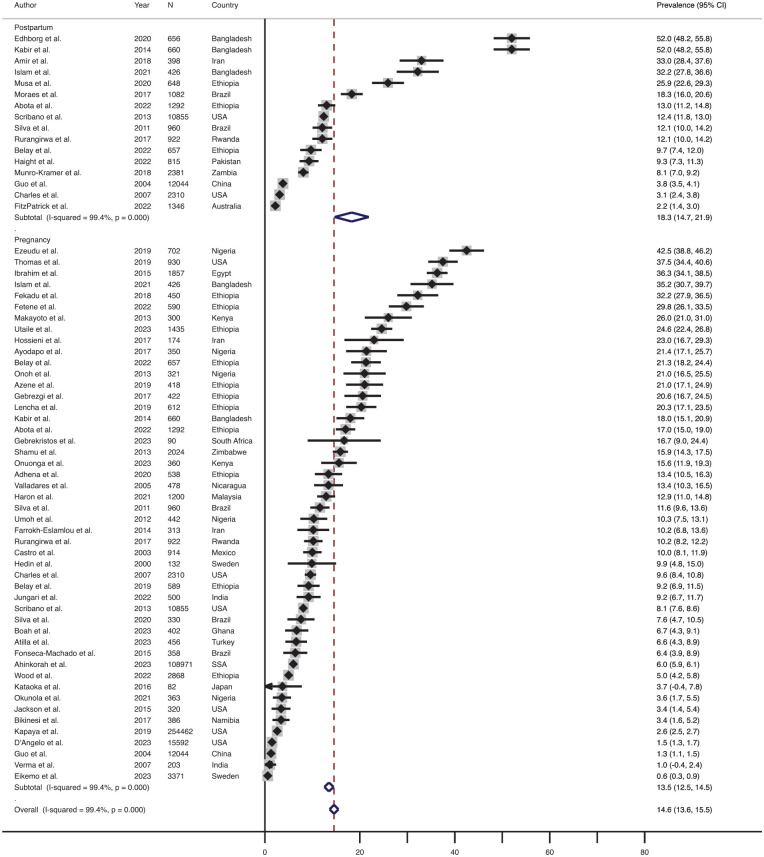
Forest plot showing the prevalence of physical IPV during pregnancy and postpartum periods. *Note*. IPV = intimate partner violence.

Out of the 55 studies, 48 (87.2%) studies measured physical IPV during pregnancy, and 16 (29.1%) articles measured physical IPV during the postpartum period. The effect size-weighted average prevalence of physical IPV during pregnancy and postpartum periods is shown in Figure 2. The effect size-weighted average prevalence of physical IPV during pregnancy was 13.5% (range: 0.6%–42.5%, *n* = 48) and during the postpartum period was 18.3% (range: 2.2%–52.0%, *n* = 16).

### Prevalence of Physical P-IPV Across Geographic Region

**Figure 3.**
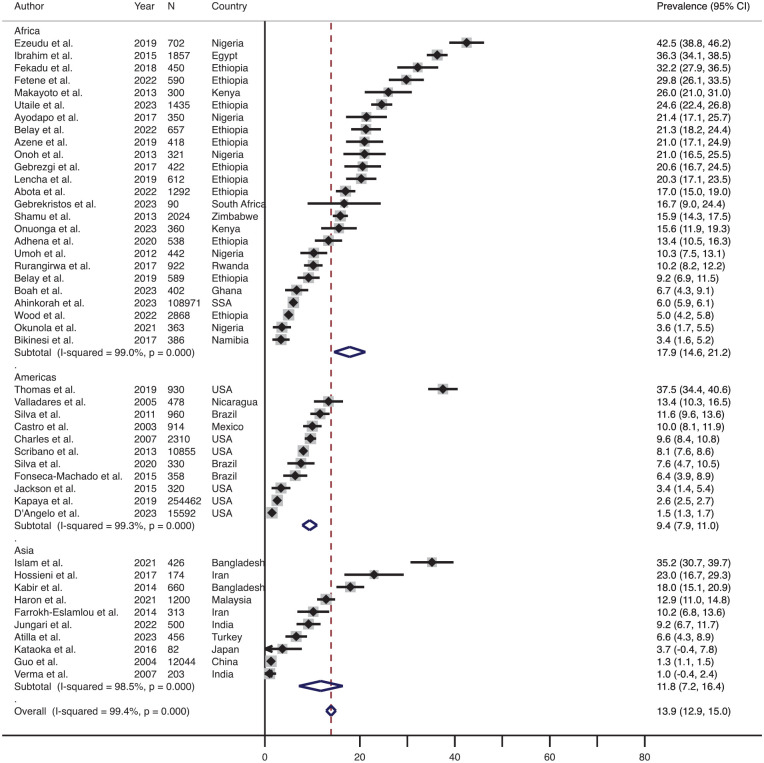
Forest plot showing the prevalence of physical IPV during pregnancy; overall and across the various regions. *Note*. IPV = intimate partner violence.

The effect size-weighted average prevalence of physical IPV during pregnancy across different geographical regions is shown in Figure 3. The prevalence in Africa was the highest (average 17.9%, range 3.4%–42.5%, *n* = 25) followed by the prevalence in Asia (average 11.8%, range 1.0%–35.2%, *n* = 10) and then in the Americas (average 9.4%, range 1.5%–37.5%, *n* = 11). The average prevalence of physical IPV during pregnancy was the lowest in Europe (average 4.9%, range 0.6%–9.9%, *n* = 2, not included in the forest plot due to the small sample size). None of the studies included in the review assessed the prevalence of physical IPV during pregnancy from the Oceania region.

**Figure 4.**
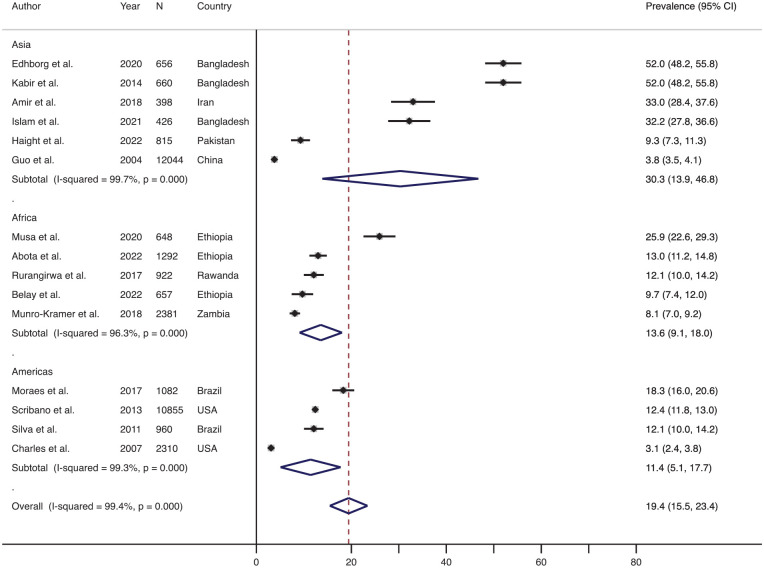
Forest plot showing the prevalence of the physical IPV during the postpartum period: overall and across the various regions. *Note*. IPV = intimate partner violence.

Similarly, Figure 4 shows the effect size-weighted average prevalence of physical IPV during the postpartum period across different geographical regions. The prevalence in Asia was the highest (average 30.3%, range 3.8%–52.0%, *n* = 6) followed by the prevalence in Africa (average 13.6%, range 8.1%–25.9%, *n* = 5) and then in the Americas (average 11.4%, range 3.1%–18.3%, *n* = 4). Only one study included in the review assessed the prevalence of physical IPV during the postpartum period (which was 2.2%) from the Oceania region, and therefore, it was not included in the forest plot. None of the studies included in the review assessed the prevalence of physical IPV during the postpartum period in Europe.

### Prevalence of Physical P-IPV Based on Socioeconomic Status

**Figure 5.**
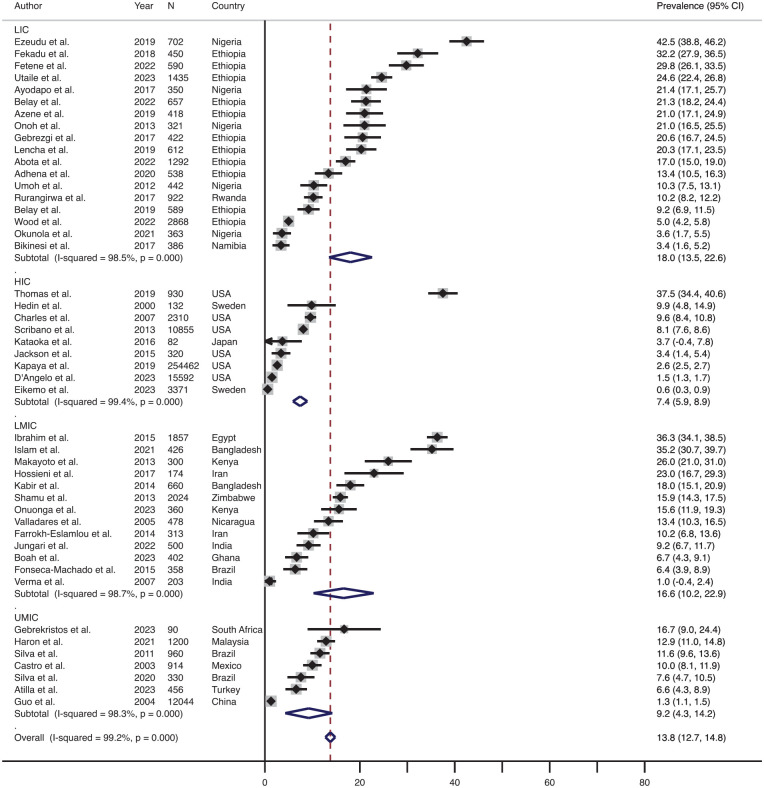
Forest plot showing the prevalence of the physical IPV during pregnancy across various socio-economical countries. *Note*. LIC = low-income countries; LMIC = lower-middle-income countries; UMIC = upper-middle-income countries; HIC = high-income countries; IPV = intimate partner violence.

Out of the 55 studies, 54 (98.2%) studies were considered to calculate the prevalence of physical IPV based on socioeconomic status because one study (Ahinkorah et al., 2023) was excluded as it included data from 16 different countries with differing levels of socioeconomic status (World-Bank, 2020). Figure 5 shows the effect size-weighted average prevalence of physical IPV during pregnancy across various countries classified based on socioeconomic status. The prevalence of physical IPV during pregnancy in low-income countries was the highest (average 18.0%, range 3.4%–42.5%, *n* = 18), which was followed by the prevalence in lower-middle income countries (average 16.6%, range 1.0%–36.3%, *n* = 13) and then in upper middle-income countries (average 9.2%, range 1.3%–16.7%, *n* = 7). The average prevalence of physical IPV during pregnancy was the lowest in the high-income countries (average 7.4%, range 0.6%–37.5%, *n* = 9).

**Figure 6.**
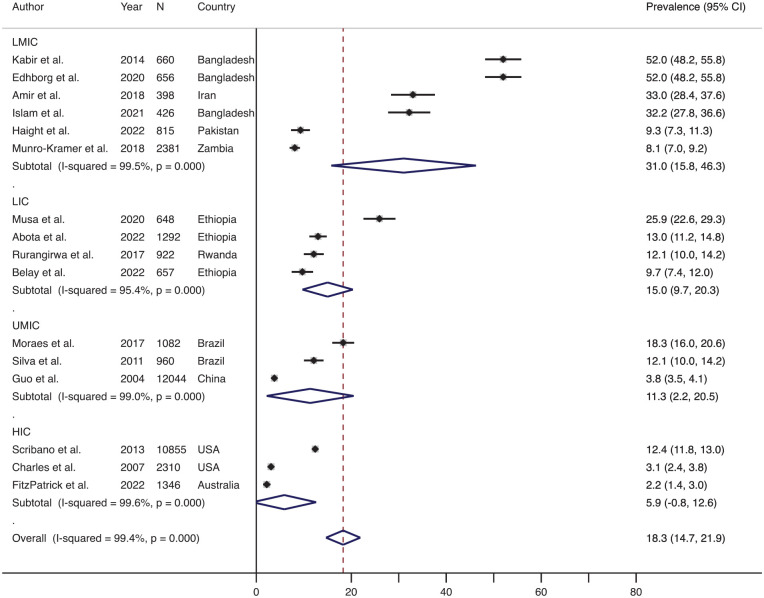
Forest plot showing the prevalence of physical IPV during the postpartum period across various socioeconomical countries. *Note*. LIC = low-income countries; LMIC = lower-middle income countries; UMIC = upper-middle income countries; HIC = high-income countries; IPV = intimate partner violence.

The effect size-weighted average prevalence of physical IPV during the postpartum period across various countries classified based on socioeconomic status is shown in Figure 6. The prevalence of physical IPV during the postpartum period in lower-middle-income countries was the highest (average 31.0%, range 8.1%–52.0%, *n* = 6), which was followed by the prevalence in low-income countries (average 15.0%, range 9.7%–25.9%, *n* = 4) and then in upper middle-income countries (average 11.3%, range 3.8%–18.3%, *n* = 3). The average prevalence of physical IPV during the postpartum period was the lowest in the high-income countries (average 5.9%, range: 2.2%–12.4%, *n* = 3).

### Prevalence of Physical P-IPV Before and During the COVID-19 Pandemic

**Figure 7.**
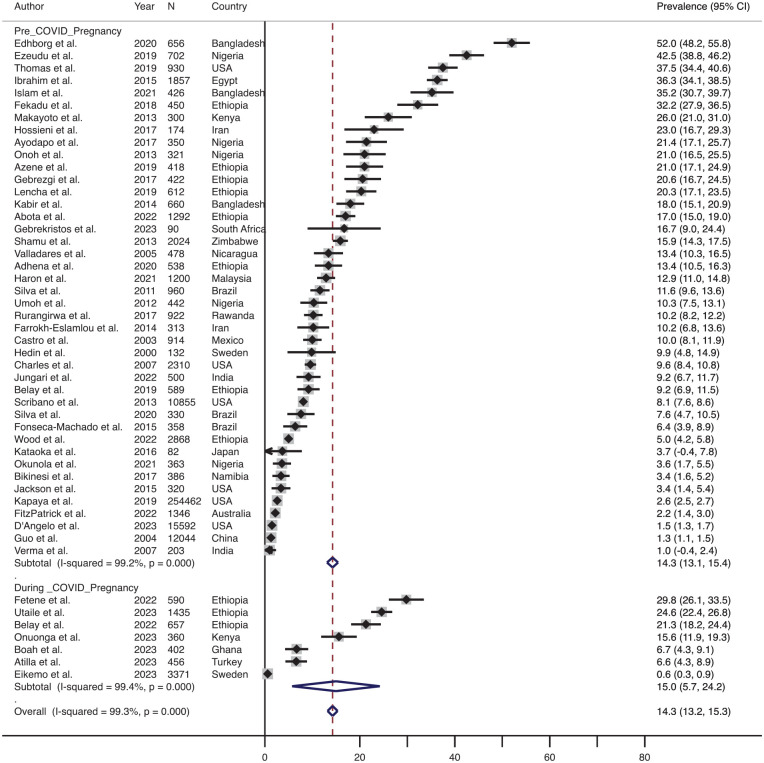
Forest plot showing the prevalence of physical IPV (intimate partner violence) before and during the COVID-19 pandemic. *Note*. IPV = intimate partner violence.

As shown in Figure 7, the effect size-weighted average prevalence of physical IPV in pregnancy before COVID-19 was 14.3% (range 1.0%–52.0%, *n* = 42) and during COVID-19 was 15.0% (range 0.6%–29.8%, *n* = 7). Similarly, the effect size-weighted average prevalence of physical IPV in the postpartum period before COVID-19 was 18.8% (range 3.1%–52.0%, *n* = 12). One article (Belay et al., 2022) included in the review assessed the prevalence of physical IPV in the postpartum period during COVID-19 (9.7%). These data were not visualized using forest plots.

## Discussion

This review provides a synthesis of the evidence relating to physical P-IPV. Post hoc objectives explored physical P-IPV and its relationship with socioeconomic classification, regions of the world, and the impacts of COVID-19. Overall, the results of this study indicate that the average sample-weighted prevalence of P-IPV across the entire perinatal period was ∼15%.

The current study further explored physical IPV separately for the pregnancy period and the postpartum period. The average sample-weighted prevalence of physical IPV during pregnancy was found to be ∼5%. The findings of the current study demonstrate a higher prevalence of physical IPV during pregnancy compared to other reviews that reported this measure – ranging from 2% to 35% (Halim et al., 2018). Furthermore, the current study results indicated that physical IPV during pregnancy is reported at similar rates as IPV (not just physical) reported by Mojahed et al. (2021). Mojahed et al. (2021) reported overall physical P-IPV prevalence rates ranging from 1.5% to 66.9%, whereas the current study reports 0.6% to 52.0%. Additionally, the results of this study indicate the average prevalence of physical IPV during the postpartum period is ∼10%. These results demonstrate the prevalence of IPV that falls within the range of other reviews that reported IPV during the postpartum period (not just physical) where overall prevalence estimates ranged from 2% to 58% (Mojahed et al., 2021). This review highlights a concerning prevalence of physical P-IPV that is similar to rates reported in previous reviews of P-IPV (not strictly physical P-IPV). These findings underscore a notably high prevalence of physical P-IPV victimization during the pregnancy and postpartum period, emphasizing the urgent need for targeted interventions and support mechanisms for individuals experiencing physical P-IPV.

### Prevalence of Physical IPV During Pregnancy Versus Postpartum Period

In both effect size- and sample-weighted estimations, physical IPV was more frequent during the postpartum period than during pregnancy. Pregnancy itself can trigger violence (Bhatta & Assanangkornchai, 2019; Carneiro et al., 2016) or reflect a continuum of previous violence (Finnbogadóttir et al., 2016). However, these results indicate that violence does not end up for child bearers at birth and is more likely to initiate, increase, or persist postpartum. Tailored efforts to prevent physical IPV after birth and support those experiencing physical IPV in the postpartum period should be pursued.

IPV during pregnancy may have direct consequences on the health of both the child bearer and unborn child. These consequences may include miscarriage, preterm birth, low birthweight, premature rupture of membranes, perinatal death, bleeding, urinary tract infections, and fetal distress among others (Pastor-Moreno et al., 2020). In addition to these physical consequences, IPV during pregnancy can also have psychological consequences on the child bearer such as mental illness, lost energy, and low self-esteem (Finnbogadóttir et al., 2016). However, non-physical forms of IPV can also cause these adverse physical and psychological effects on both the child bearer and unborn child (Pastor-Moreno et al., 2020). Thus, it is important not to discredit the independent or compounding detriments of all forms of IPV in the context of health outcomes during the pregnancy and postpartum period as physical IPV rarely occurs independently. Deciphering separate effects of sub-categories of IPV during both the pregnancy and postpartum periods is likely to be extremely complex and important.

### Prevalence of Physical P-IPV Across Geographic Region

The results of this study revealed that the prevalence of IPV during pregnancy is the highest in Africa, followed by Asia, the Americas, and Europe. Similarly, the prevalence rate during the postpartum period is the highest in Asia followed by Africa, the Americas, and Europe. This could be due to complexities related to socioeconomical status, age, decision-making capacity, marital power dynamics, and factors related to gender, childhood trauma, substance use, and/or depression (Ahinkorah et al., 2023; Fulu et al., 2013). In light of our findings indicating regional disparities in the prevalence of IPV, with notably higher rates observed in Africa and Asia compared to the Americas and Europe, it becomes apparent that contextual factors may contribute to these variations. These factors underscore the need for targeted interventions that address the specific sociocultural and economic contexts of each region to effectively mitigate P-IPV and to support affected child bearers.

### Prevalence of Physical P-IPV Based on Socioeconomic Classification

In terms of the impact of socioeconomic status, the current study reveals the prevalence of physical IPV during pregnancy in low-income countries is the highest while the average prevalence is the lowest in the high-income countries. Overall, during pregnancy, the prevalence rates of IPV in low-income and lower-middle-income countries are approximately 2.5 times and 2 times more than that of the prevalence in higher-income countries. Coll et al. (2020) demonstrated inequalities in IPV prevalence across socioeconomic status particularly from low-income and middle-income countries through an examination of 46 countries’ health survey data. Furthermore, research has demonstrated that low socioeconomic status increases the risk of experiencing IPV (Abramsky et al., 2011). Our findings are consistent with the published literature on IPV (Abramsky et al., 2011; Coll et al., 2020). This is not surprising as socioeconomic status is widely recognized as one of the key social determinants of health and may be attributed to various socioeconomic factors, including poverty, limited access to education, employment opportunities, and healthcare services (Glymour et al., 2014; Marmot & Wilkinson, 2005). In low-income countries, economic instability and lack of social support may exacerbate stressors, increasing the likelihood of conflict within intimate relationships during pregnancy.

Cultural norms and attitudes toward IPV also play a pivotal role, with societies that normalize or condone violence inhibiting individuals from seeking help or disclosing their experiences (Murvartian et al., 2023). Conversely, progressive attitudes toward gender equality and zero-tolerance policies on violence in high-income countries empower individuals to report instances of P-IPV and access support services. Moreover, variations in healthcare infrastructure and support services further contribute to disparities, with limited resources and trained professionals hindering early detection and intervention efforts in low-income countries. Intersectionality further compounds these disparities, as marginalized populations within both low-income and high-income countries face unique barriers to accessing support services due to intersecting forms of discrimination and stigma. Efforts to address P-IPV must be comprehensive, intersectional, and tailored to meet the diverse needs of vulnerable populations, considering the adverse effects on maternal and child health outcomes.

### Prevalence of Physical P-IPV Before and During the COVID-19 Pandemic

While these results show there was no marked difference in the prevalence of physical IPV in pregnancy before and during COVID-19, previous work has shown there was an increase in IPV amid the COVID-19 pandemic (Uzoho et al., 2023). While an increase in IPV was noted worldwide (Bouillon-Minois et al., 2020; Brink et al., 2021), the results of the current study showed the prevalence of physical P-IPV before COVID-19 was much higher than during COVID-19. These conflicting results and decreased prevalence estimates of P-IPV may be partly due to under-reporting resulting from the stay-at-home orders put in place to curtail the spread of the pandemic, causing victims to be less likely to seek help (Uzoho et al., 2023). Among the adverse effects of the restrictive COVID-19 measures were increased IPV and negative influences on women’s access to health care, social service, and social support (Decker et al., 2022; Wenham et al., 2020). Factors related to the pandemic that contributed to increased rates of IPV include isolation, economic hardship, increased opportunity for the perpetrator to control behaviors, and other physical and emotional challenges. Additionally, many community facilities designed to help victims of IPV were closed or only offered limited services during the pandemic, making it difficult for women to seek assistance and increasing their vulnerability to violence by their intimate partners (Uzoho et al., 2023).

## IPV and BI Link

IPV-BI is a well-documented outcome following physical IPV (Esopenko et al., 2021; Haag et al., 2022; Valera & Berenbaum, 2003). IPV-BI, most commonly occurring among women, is theorized to differ from traditional mechanisms (e.g., sports, traffic incidents) of traumatic brain injury. The majority of concussion and BI research has been conducted among male athletes and in male animal models. Thus, if the neurometabolic cascade and subsequent pathophysiology differ among males and females, and how varying hormonal differences impact this pathophysiology remains to be elucidated (Haynes & Goodwin, 2023). Further, IPV is commonly cyclic and escalating (Krug et al., 2002), further differentiating the potential BIs that result from traditional single-event mechanisms. For example, a professional athlete will commonly sustain a single BI during sport from one isolated event, be removed from play, have a team of medical personnel assess, treat, and care for them, all with the goal of successful rehabilitation and recovery without any stigma, financial stress, housing stress, etc. Conversely, a survivor of IPV may sustain repeated BIs over time, not access medical, social, or supportive resources, and continue to parent, work, and survive in the violent situation under the constraints of stigma, financial instability, housing instability, social scrutiny, etc. Thus, two different trajectories may exist for what are fundamentally two different injuries. Short-term, long-term, and chronic effects of IPV are well documented to affect every bodily system (Black, 2011; Black et al., 2011; Campbell, 2002), but investigation of the specific nature and detriment of IPV-BI has not been fully uncovered. When applying this concept to the population during the pregnancy and postpartum period, it is probable that the vast majority of physical P-IPV survivors sustain BIs at the hands of their intimate partners. There is ongoing debate that gonadal hormones and fluctuating hormones during pregnancy may affect the susceptibility to and prognosis following BI (Berry et al., 2011; Haldar et al., 2023; Haynes & Goodwin, 2023; Hoekzema et al., 2022). However, this is a debated topic with no uniform conclusion and the extent of child bearer and fetal neuroprotection is unknown. Additionally, IPV-BI is often the result of uni- or multi-modal mechanisms of assault. As previously mentioned, IPV-BI commonly occurs from a direct blow, indirect force, and/or NFS (Esopenko et al., 2021; Valera & Berenbaum, 2003). NFS ceases or impairs the flow of air and/or blood to and/or from the brain, making hypometabolic injury likely (Messing et al., 2018; Pritchard et al., 2017; White et al., 2021). In the framing of IPV-BI during the pregnancy and postpartum period, this means both the child bearer and fetus are at risk of hypometabolic injury from NFS in addition to the neuropathological sequelae from direct or indirect trauma (Messing et al., 2018). Finally, intimate partner homicide is common during IPV, with risks increasing exponentially when a survivor is attempting to leave a violent relationship and when there is a history of NFS (Campbell et al., 2007; Glass et al., 2008).

The intersection of P-IPV and IPV-BI highlights a dire need for comprehensive screening, universal education, and intervention strategies that are sensitive to the nuances of health during the pregnancy and postpartum period. Such strategies must account for the dual risk of child bearers and fetal harm in the scope of physical and neurological damage. Moreover, the potential for IPV-BI to impair cognitive and emotional functioning further complicates the ability of childbearing individuals to seek help and navigate safety planning, underscoring the urgency of integrating targeted support mechanisms within frameworks of care during the pregnancy and postpartum period. Preventative measures, healthcare provision, and support systems must be adequately equipped to address the complex needs of IPV survivors, including potential IPV-BI. This integration aims to mitigate the immediate and long-term consequences of IPV-BI, fostering a safer and more supportive environment for healing and resilience during and after the pregnancy and postpartum period.

### Limitations

All methods used to summarize data from heterogeneous physical P-IPV studies yield limitations. The considerable variation of prevalence estimates found was indicative of considerable between-study variation. Definitions of IPV, physical IPV, postpartum period, methods, and time of measurement differed substantially across the studies included in this review. These factors may have affected the prevalence estimates of physical P-IPV. Both the sample- and effect size-weighted average methods have hindrances and must be interpreted cautiously. As with all studies of the prevalence of IPV, inherent limitations surrounding measurement and reporting exist. It is likely that the estimates reported in this review are underestimates. More robust efforts to capture P-IPV prevalence should be pursued. The majority of P-IPV prevalence studies focus on cisgender, heterosexual women who experience violence from partners who are men. Many research gaps exist surrounding IPV and IPV-BI in sexual and gender minority communities. Findings from this study may not be applicable to more diverse settings and circumstances. As the primary objective was to assess the prevalence of physical P-IPV not P-IPV-BI, studies that met the inclusion criteria did not permit the examination of P-IPV-BI. Thus, the prevalence of P-IPV-BI warrants further investigation that is beyond the scope of this review.

## Recommendations for Future Research, Generalizability, and Practical Implications

Several recommendations for future research and practical implications were mentioned in the discussion section. The major points are summarized in Tables 2 and 3.

**Table 2.** Implications of the Review for Practice, Policy, and Research.

Implications for practice	• We identified a high rate of prevalence of physical IPV during pregnancy and the postpartum period. Thus, it is important to consider the screening consequences of physical IPV in this population. • Based on the literature on the general population, there could be a strong linkage between physical IPV and BI. Therefore, screening of IPV-BI in victims of physical IPV during pregnancy and the postpartum period is necessary.
Implications for policy	• Our review can inform policymakers to invest in the early detection, awareness, and appropriate management of IPV-BI in the pregnant and postpartum population.
Implications for research	• Further research is required to explore the true prevalence of physical IPV, its linkage with BI and immediate as well as longer-term impacts of IPV as well as IPV-BI in the pregnant and postpartum population. • Research on early detection and effective management of IPV-BI in the pregnant and postpartum population is warranted to improve their quality of life and to minimize its long-term consequences.

*Note*. IPV = intimate partner violence; BI = brain injury; IPV-BI = intimate partner violence-caused brain injury.

**Table 3.** Critical Findings of the Systematic Review.

Critical findings
• Overall prevalence of physical IPV during pregnancy and post-partum period is approximately 15%, which appears to have increased since 2020. • Although various factors such as geographical regions, and socioeconomic variance contribute, physical IPV during pregnancy and post-partum period is still pervasive across all lands and statuses. • Future research that directly examines an etiological link between physical IPV during pregnancy and post-partum period and brain injury should be undertaken.

*Note*. IPV = intimate partner violence.

This review included articles that were conducted in various geographical regions in which heterogenous participants in terms of various characteristics such as culture, beliefs, ethnicity, socioeconomical status, etc. were recruited. In addition to the overall prevalence of the study, prevalence based on geographical regions, socioeconomical status, and COVID-19 pandemic were also presented. Moreover, the data analysis and results synthesis and discussion were conducted by a research team having varying characteristics related but not limited to age, sex, gender, ethnicity, visible minority, culture, education, and experience. Therefore, these features of the review would increase the generalizability and implications of the findings of this review.

## Conclusion

With an overall prevalence of ∼15%, physical P-IPV remains a looming and growing threat to child bearers, fetal, and early childhood health. Physical P-IPV results in short- and long-term pathophysiological health detriments for the child bearer, fetus, and child. Although regional and socioeconomic variance exists, physical P-IPV is still pervasive across all lands and statuses. Further, IPV rates have risen since COVID-19, and, although preliminary, physical P-IPV appears to have increased since 2020. Given the >80% prevalence of IPV-BI among survivors of physical IPV, further research that directly examines the possibility that BI is also occurring in the population during the pregnancy and postpartum period should be undertaken to uncover the true prevalence and impact of P-IPV-BI, prevent P-IPV, and improve the response to P-IPV and P-IPV-BI.

## Supplemental Material

sj-docx-1-tva-10.1177_15248380241309292 – Supplemental material for The Prevalence of Physical Intimate Partner Violence During Pregnancy and the Postpartum Period: A Systematic Review With Implications for Probable Violence-Caused Brain Injury Among Child Bearers Supplemental material, sj-docx-1-tva-10.1177_15248380241309292 for The Prevalence of Physical Intimate Partner Violence During Pregnancy and the Postpartum Period: A Systematic Review With Implications for Probable Violence-Caused Brain Injury Among Child Bearers by Shambhu P. Adhikari, Tori N. Stranges, Bradi R. Lorenz, Rory A. Marshall, Nelson Jiang and Paul van Donkelaar in Trauma, Violence, & Abuse
