# Supplementary material for: The Prevalence of Physical Intimate Partner Violence During Pregnancy and the Postpartum Period: A Systematic Review With Implications for Probable Violence-Caused Brain Injury Among Child Bearers
Source: Trauma Violence Abuse. 2025 Jan 2;27(2):275–93. doi: 10.1177/15248380241309292 (PMC12953689; doi:10.1177/15248380241309292)
Supplement: sj-docx-1-tva-10.1177_15248380241309292 – Supplemental material for The Prevalence of Physical Intimate Partner Violence During Pregnancy and the Postpartum Period: A Systematic Review With Implications for Probable Violence-Caused Brain Injury Among Child Bearers [file sj-docx-1-tva-10.1177_15248380241309292.docx]

**Supplementary Table 1:** Search strategies (Medline and Embase)

|  | **Medline (Via. Ovid)** | **Embase (Via. Ovid)** |
| --- | --- | --- |
| P | 1. perinatal period/ | 1. peripartum period/ or postpartum period/ or pregnancy/ or perinatal period/ or prenatal period/ or postnatal period/ |
|  | 1. puerperium/ | 1. Pregnant Women/ |
|  | 1. (Perinatal or postpartum or puerperium or peripartum or pregnancy or pregnant).mp. [mp=title, abstract, heading word, drug trade name, original title, device manufacturer, drug manufacturer, device trade name, keyword heading word, floating subheading word, candidate term word] | 1. (perinatal or peripartum or postpartum or pregnancy or pregnant or puerperium).mp. [mp=title, abstract, heading word, drug trade name, original title, device manufacturer, drug manufacturer, device trade name, keyword heading word, floating subheading word, candidate term word] |
|  | 1. pregnancy/ | 1. 1 OR 2 OR 3 |
|  | 1. 1 OR 2 OR 3 OR 4 |  |
| I | 1. partner violence/ or domestic violence/ or marital rape/ | 1. intimate partner violence/ or spouse abuse/ |
|  | 1. (abuse or violence) adj3 (partner or spousal or wife or spouse or domestic)) or IPV).mp. [mp=title, abstract, heading word, drug trade name, original title, device manufacturer, drug manufacturer, device trade name, keyword heading word, floating subheading word, candidate term word] | 1. ((abuse or violence) adj3 (domestic or partner or spouse or spousal or wife)).mp. [mp=title, abstract, heading word, drug trade name, original title, device manufacturer, drug manufacturer, device trade name, keyword heading word, floating subheading word, candidate term word] |
|  | 1. battered woman/ | 1. Battered Women/ |
|  | 1. ((battered adj3 (wom#n or wife or spouse or spousal)).mp. [mp=title, abstract, heading word, drug trade name, original title, device manufacturer, drug manufacturer, device trade name, keyword heading word, floating subheading word, candidate term word] | 1. ((battered adj3 (wom#n or wife or spouse)).mp. [mp=title, abstract, heading word, drug trade name, original title, device manufacturer, drug manufacturer, device trade name, keyword heading word, floating subheading word, candidate term word] |
|  | 1. 6 OR 7 OR 8 OR 9 | 1. 5 OR 6 OR 7 OR 8 |
|  | 1. 5 AND 10 | 1. 4 AND 9 |
| O | 1. prevalence/ | 1. prevalence/ |
|  | 1. prevalence*.mp. | 1. Prevalence*.mp. |
|  | 1. 12 OR 13 | 1. 11 OR 12 |
|  | 1. 11 AND 14 | 1. 11 AND 13 [PIO] |
|  | 1. limit 15 to (english language and yr="2000 -Current") | 1. limit 14 to (english language and yr="2000 -Current") |

Note: P: Population, I: Issue, O: Outcome. Since the “comparator” is not applicable in observation studies, the PIO model (modification of PICO) was considered.

**Supplementary Table 2:** Search strategies (CINAHL and PsycINFO)

|  | **CINAHL (Via. EBSCO)** | **PsycINFO (Via. EBSCO)** |
| --- | --- | --- |
| P | S1. (MH "Perinatal Period") | S1. DE "Perinatal Period" OR DE "Neonatal Period" OR DE "Antepartum Period" OR DE "Postnatal Period" |
|  | S2. Peripartum OR Peripartum Period OR Perinatal OR Postnatal OR Postpartum OR Pregnancy OR Pregnant | S2. Peripartum OR Postpartum OR Puerperium OR Peripartum OR Pregnancy OR Pregnant |
|  | S3. S1 OR S2 | S3. S1 OR S2 |
| I | S4. (MH "Intimate Partner Violence") OR (MH "Domestic Violence+") OR (MH "Gender-Based Violence") | S4. DE "Domestic Violence" OR DE "Intimate Partner Violence" OR DE "Physical Abuse" OR DE "Battered Females" |
|  | S5. Dating violence OR Dating abuse OR Elder violence OR Elder abuse OR Partner abuse OR Partner violence | S5. Dating violence OR Partner violence OR Partner abuse OR Elder abuse OR Elder violence OR Dating abuse OR Gender Violence OR Physical Abuse OR battered women |
|  | S6. ((abuse or violence) n3 (partner or spouse or spousal or wife or domestic) | S6. ((abuse or violence) n3 (partner or spouse or spousal or wife or domestic)) |
|  | S7. S4 OR S5 OR S6 | S7. S4 OR S5 OR S6 |
|  | S8. S3 AND S7 | S8. S3 AND S7 |
| O | S9. (MH "Prevalence") or prevalence* | S9. DE “prevalence” |
|  | S10. S8 AND S9 | S10. S8 AND S9 |
|  | Limit: English language and Year: Jan 2000 to July 2023 | Limit: English language and Year: Jan 2000 to July 2023 |

Note: P: Population, I: Issue, O: Outcome. Since the “comparator” is not applicable in observation studies, the PIO model (modification of PICO) was considered.

**Supplementary Table 3:** Description of physical IPV based on different tools used to assess violence

| **Tools used to assess violence** | **# of studies (N= 55)** | **Description of physical IPV** |
| --- | --- | --- |
| World Health Organization (WHO) Multicountry Study | 27 | “Have you experienced any of the following during the current pregnancy: (a) slapped you or threw something at you that could hurt you; (b) pushed you or shoved you; (c) hit you with his fist or with something else that could hurt you; (d) kicked you, dragged you, or beat you up; (e) choked you or burnt you on purpose; and (f) threatened to use or used a gun, knife, or other weapons against you.” |
| Revised Conflict Tactic Scale- 2 (Revised CTS-2) | 5 | ‘Push you, shake you or throw something at you;’ ‘slap you;’ ‘twist your arm or pull your hair;’ ‘punch you with his fist or with something that could hurt you;’ ‘kick you, drag you or beat you up;’ ‘try to choke you or burn you on purpose;’ ‘threaten or attack you with a knife, gun or other weapon’ |
| Abuse Assessment Screen (AAS) | 3 | “Since you became pregnant, have you been punched, kicked, pushed, or injured by your partner or someone else important to you?”, “Since you became pregnant, have you been subjected to psychological violence such as verbal abuse, threats, or coercion by your partner or someone else important to you?” and “Since you became pregnant, have you been subjected to sexual acts against your will by your partner or someone else important to you?”. |
| Measure created for Study | 8 | NA |
| Pregnancy Risk Assessment Monitoring System (PRAMS) | 3 | ‘‘During the 12 months before you got pregnant with your new baby, did your husband or partner push, hit, slap, kick, choke, or physically hurt you in any other way?’’ and ‘‘During your most recent pregnancy, did your husband or partner push, hit, slap, kick, choke, or physically hurt you in any other way?.’’ |
| Women's Health and Life Experiences Questionnaire | 1 | Physical violence defined as when a woman reported that she had been slapped, pushed or shoved, hit with fist, kicked, dragged or beaten, choked, burnt, or threatened to use or used a weapon by her partner. |
| Composite Abuse Scale | 1 | “Pushed, grabbed, or shoved me” |
| Severity of Violence Against Women Scale (SVAW) | 1 | Has one of the following things happened to you after giving birth?  Symbolic violence, e.g. threw an object at her; threats of mild violence, e.g. shook a fist at her; threats of moderate violence, e.g. threatened to destroy property; threats of serious violence, e.g. threatened her with a knife or gun. Acts of violence such as: mild violence, e.g. shook or roughly handled her; minor violence, e.g. spanked her; moderate violence, e.g. slapped her around her face and head; serious violence, e.g. used a knife or gun on her |
| NorVold Domestic Abuse Questionnaire (NORAQ) | 1 | Physical violence defined as use of physical force or weapons in attacks that injured or harmed a woman, including beating, kicking, pulling hair, biting, burning, attacks with weapons and objects, and murder |
| Ongoing Abuse Screen (OAS) | 1 | Are you presently emotionally or physically abused by your partner or someone important to you? Are you presently being hit, slapped, kicked, or otherwise physically hurt by your partner or someone important to you? Are you presently being forced to have sexual activities? Are you afraid of your partner or anyone of the following (circle if applicable): (husband/wife), (ex-husband/ex-wife), (boyfriend/girlfriend), stranger. (If pregnant) Have you been hit, slapped, kicked, or otherwise physically hurt by your partner or someone important to you during pregnancy? |
| Index of Spouse Abuse (ISA) | 1 | Physical (“My partner punches me with his fists”) aggression. |
| Intimate Partner Violence During Pregnancy Instrument (IPVPI) | 1 | While determining physical violence, pregnant women were asked whether they had been exposed to the following behaviors by their partners: (1) slapping, hitting, kicking, (2) punching, (3) pushing, pulling hair, (4) hitting with any object, beating, (5) hurting with a sharp/penetrating tool, (6) covering her mouth with his hand and (7) hostile physical advance. |
| The Future of Families and Child Wellbeing Study (FFCWS) questionnaire | 1 | To measure physical violence, both parents reported how frequently (often, sometimes, or never) they had been hit or slapped during their relationship. Because even sometimes hitting or slapping a partner is significant, we dichotomized this variable to indicate any (i.e., often or sometimes) physical violence. |
| Measure Not Mentioned | 1 | NA |
